# Platelet-rich fibrin prepared from stored whole-blood samples

**DOI:** 10.1186/s40729-017-0068-4

**Published:** 2017-03-01

**Authors:** Kazushige Isobe, Masashi Suzuki, Taisuke Watanabe, Yutaka Kitamura, Taiji Suzuki, Hideo Kawabata, Masayuki Nakamura, Toshimitsu Okudera, Hajime Okudera, Kohya Uematsu, Koh Nakata, Takaaki Tanaka, Tomoyuki Kawase

**Affiliations:** 1 Tokyo Plastic Dental Society, Kita-ku, Tokyo, Japan; 2 Division of Oral Implantology, Niigata University Medical and Dental Hospital, Niigata, Japan; 3 Bioscience Medical Research Center, Niigata University Medical and Dental Hospital, Niigata, Japan; 4 Department of Materials Science and Technology, Niigata University, Niigata, Japan; 5 Division of Oral Bioengineering, Institute of Medicine and Dentistry, Niigata University, Niigata, Japan

**Keywords:** Platelet-rich fibrin, Coagulation, Fibrin fiber, Anticoagulant, Calcium chloride

## Abstract

**Background:**

In regenerative therapy, self-clotted platelet concentrates, such as platelet-rich fibrin (PRF), are generally prepared on-site and are immediately used for treatment. If blood samples or prepared clots can be preserved for several days, their clinical applicability will expand. Here, we prepared PRF from stored whole-blood samples and examined their characteristics.

**Methods:**

Blood samples were collected from non-smoking, healthy male donors (aged 27–67 years, *N* = 6), and PRF clots were prepared immediately or after storage for 1–2 days. Fibrin fiber was examined by scanning electron microscopy. Bioactivity was evaluated by means of a bioassay system involving human periosteal cells, whereas PDGF-BB concentrations were determined by an enzyme-linked immunosorbent assay.

**Results:**

Addition of optimal amounts of a 10% CaCl_2_ solution restored the coagulative ability of whole-blood samples that contained an anticoagulant (acid citrate dextrose) and were stored for up to 2 days at ambient temperature. In PRF clots prepared from the stored whole-blood samples, the thickness and cross-links of fibrin fibers were almost identical to those of freshly prepared PRF clots. PDGF-BB concentrations in the PRF extract were significantly lower in stored whole-blood samples than in fresh samples; however, both extracts had similar stimulatory effects on periosteal-cell proliferation.

**Conclusions:**

Quality of PRF clots prepared from stored whole-blood samples is not reduced significantly and can be ensured for use in regenerative therapy. Therefore, the proposed method enables a more flexible treatment schedule and choice of a more suitable platelet concentrate immediately before treatment, not after blood collection.

## Background

Blood preservation is generally and widely used in the fields of blood transfusion and surgery for either autologous or allogeneic blood [1–3]. In case of small lots of blood-derived materials used in regenerative therapy, such as platelet concentrates, it is generally accepted that autologous blood samples should be collected on-site and immediately centrifuged for processing [4]. Accordingly, it is officially recommended to use thus prepared materials immediately. The advantages of this preparation protocol are the zero cost of preservation and no risk of degradation and contamination.

In Niigata University Hospital, when relatively severe surgical operations (e.g., large bone defects that require hospitalization for alveolar ridge augmentation and sinus floor elevation) are planned, relatively large volumes of blood samples are usually collected the day before the operation, and platelet-rich plasma (PRP) is prepared and stored at ambient temperature until use [5]. Nevertheless, there are no established methods for preparation of self-clotted platelet concentrates from stored whole-blood (WB) samples. This may be another reason why platelet-rich fibrin (PRF) should be prepared on-site and used immediately.

On the other hand, if PRF can be prepared from stored WB samples on the next day or later without significant reduction in the bioactivity, clinical applications of PRF will expand. In this study, we developed a method for preparation of PRF from stored WB samples by adding CaCl_2_ and evaluated the quality in terms of suitability for regenerative therapy. As a result, we successfully validated the method and ensured the quality of PRF prepared from stored WB samples.

## Methods

### Blood collection, preservation, and PRF preparation

The study design and consent forms for all procedures performed on the study subjects were approved by the ethics committee for human subjects at Niigata University School of Medicine in accordance with the Helsinki Declaration of 1975 as revised in 2008.

With informed consent, blood samples (~9.0 mL per tube) were collected from six non-smoking, healthy, male volunteers (27 to 67 years old) using 21-gauge needles equipped with conventional vacuum plain glass tube (Plain BD Vacutainer Tube; Becton, Dickinson and Company, Franklin Lakes, NJ, USA) as described previously [6–8]. For preparation of control PRF by the conventional method, the anticoagulant was not added. Blood samples were immediately centrifuged or stored by gentle mixing using a tube rotator at ambient temperature (18–22 °C).

The blood samples collected with the anticoagulant and stored for up to 2 days were centrifuged by means of a Medifuge centrifugation system (Silfradent S.r.l., Santa Sofia, Italy). After elimination of the red blood cell fractions, the resulting PRF clots, more specifically termed as concentrated growth factors (CGF) [9], were stored at −80 °C until measurement of growth factor concentration.

For preparation of platelet-poor plasma (PPP), blood samples (~9.0 mL) were collected from the same volunteers by means of plastic vacuum blood collection tubes (Neotube®; NIPRO, Osaka, Japan) equipped with 21-gauge needles, in the presence of 1.0 mL acid citrate dextrose solution-A formulation (ACD-A; Terumo, Tokyo, Japan), an anticoagulant [8, 10]. The blood samples were centrifuged on a KS-5000 centrifuge (Kubota, Tokyo, Japan) equipped with a swing rotor at 1700 rpm (530*g*) and 3000 rpm (1660*g*) for the first and second spin (8 min), respectively. The resulting supernatant fractions were collected as PPP preparations. To form fibrin clots, bovine thrombin (Liquid Thrombin MOCHIDA softbottle, Mochida Pharmaceutical Co., Ltd., Tokyo, Japan) was added to PPP at a final volume percentage of 2.5%.

### Determination of glucose, calcium, and pH

WB samples were quickly centrifuged at 1500 rpm for 3 min to prepare plasma fraction, which were subjected to determine total free calcium levels using a commercial kit based on MXB method (Calcium E-test WAKO; Wako Pure Chemicals, Osaka, Japan).

Stored WB samples were then mixed intermittently with 200 μL (20 μL × 10 times) of 10% CaCl_2_ solution and centrifuged by a Medifuge centrifugation system to prepare PRF. When lower amounts of CaCl_2_ were added, PRF clots were less reproducibly prepared. When higher amounts of CaCl_2_ were added intermittently, or when the optimal amount of CaCl_2_ were added at once, PRF clots were never prepared (Kawase, unpublished observations).

The supernatant serum fractions were subjected to determine calcium and glucose levels as described above and using a commercial kit based on GOD method (Glucose CII-test WAKO; Wako Pure Chemicals). The serum fractions were also used to determine pH levels by pH indicators (MColorHast; EMD Millipore Corp., Billerica, MA, USA).

### A bioassay on human periosteal cells

The frozen PRF samples were minced with scissors and homogenized using a disposable homogenizer (BioMasher II, Nippi, Tokyo, Japan). After high-speed centrifugation (7340*g*), supernatants (PRF extracts) were collected and used for the bioassay described below and for measurement of growth factor levels.

Because alveolar periosteum strongly contributes to regeneration of periodontal skeletal tissue [11], we used human alveolar bone-derived periosteal cells for evaluation of the potency and efficacy of PRF preparations. The periosteal cells were obtained and expanded as described elsewhere [8, 12]. With informed consent, human periosteum tissue segments were aseptically excised from the periodontal tissue on the healthy buccal side of the retromolar region of the mandibles of two non-smoking female volunteers (age = 19 and 29). Small periosteum pieces were expanded to form multilayered cellular periosteal sheets (∅ 30–40 mm), and then these sheets were enzymatically digested with 0.05% trypsin plus 0.52 mM EDTA (Invitrogen, Carlsbad, CA, USA) to release single cells. After expansion in monolayer cultures, the cells were seeded at a density of 0.4 × 10^4^ per well in 24-well plates and treated with PRF extracts (0, 0.5, 1, 2, or 4%) for 72 h in DMEM containing 1% of fetal bovine serum (Invitrogen, Carlsbad, CA, USA). Six different lots of PRF extracts were used for each experiment. At the end of the incubation periods, the cells were harvested using 0.05% trypsin plus 0.53 mM EDTA and immediately counted on an automated cell counter (Moxi-z; ORLFO Technologies, Ketchum, ID, USA) (*N* = 6) [13].

### Quantification of a growth factor by an enzyme-linked immunosorbent assay (ELISA)

PRF extracts prepared as described above were subjected to measurement of PDGF-BB levels using the Human PDGF-BB Quantikine ELISA Kit (R&D Systems, Inc., Minneapolis, MN, USA) as described previously [8].

### Scanning electron microscopy (SEM)

The PRF clots that were compressed in a stainless-steel compressor were fixed with 2.5% neutralized glutaraldehyde, dehydrated with a series of ethanol solutions and *t*-butanol, freeze-dried, and then examined under a scanning electron microscope (TM-1000, Hitachi, Tokyo, Japan) with an accelerating voltage of 15 kV, as described elsewhere [7, 14].

### Statistical analysis

The data were expressed as mean ± standard deviation (SD). For multigroup comparisons, statistical analyses were conducted to compare the mean values by one-way analysis of variance (ANOVA) followed by Tukey’s multiple-comparison test (SigmaPlot 12.5; Systat Software, Inc., San Jose, CA, USA). Differences with *P* values <0.05 were considered significant.

## Results

**Fig. 1:**
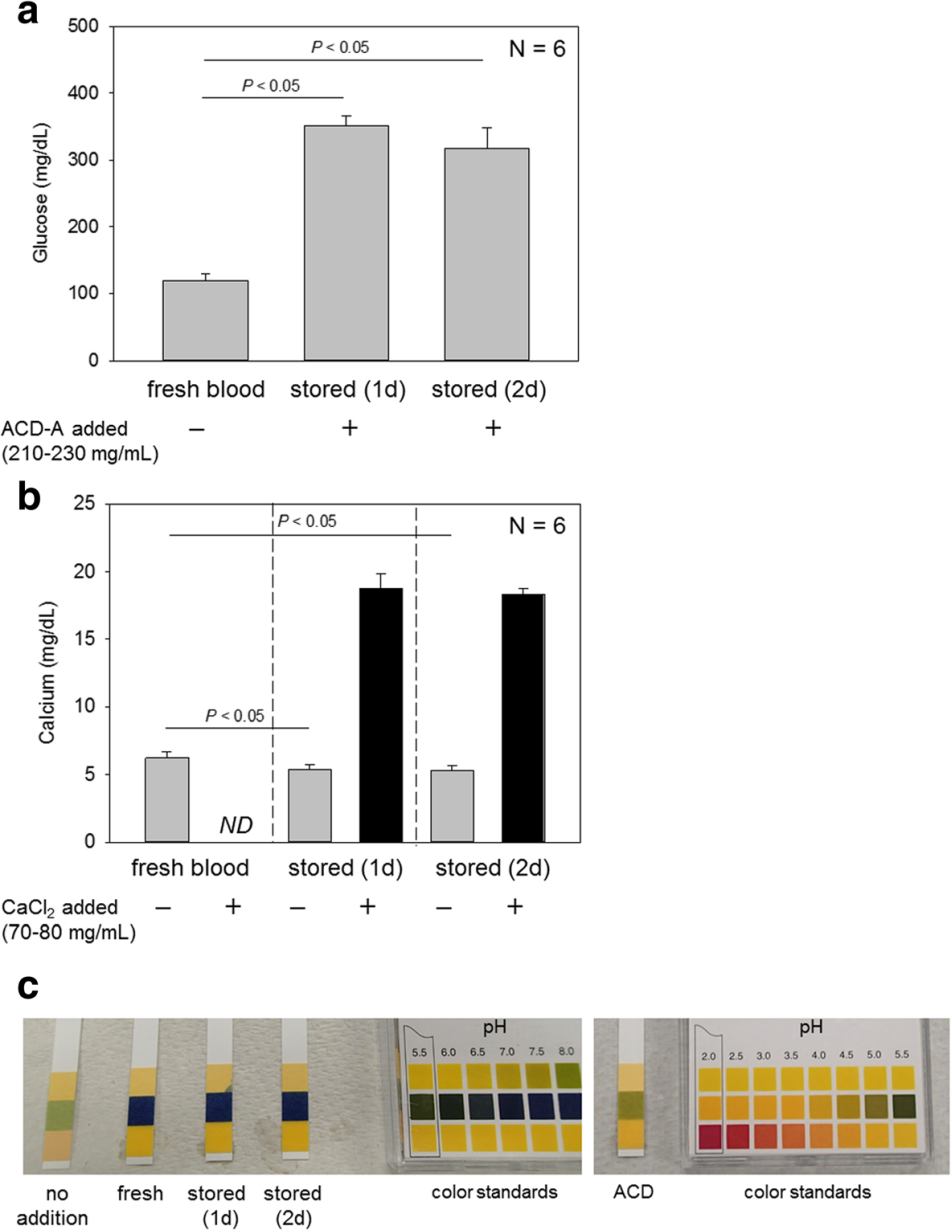
Glucose levels (**a**), calcium levels (**b**), and pH (**c**) of stored WB samples. Supernatant serum fractions were examined. Plasma fractions prepared by quick centrifugation were used to determine calcium levels in fresh and stored WB samples that were not added CaCl_2_. *N* = 6

Glucose and calcium contents and pH of WB or serum samples after centrifugation are shown in Fig. 1. Because glucose is contained in the ACD-A solution, glucose levels in the stored WB and serum samples (see Fig. 4c) after centrifugation were significantly greater than those of freshly collected WB samples. Total free calcium levels, including calcium chelated by citrate, in WB samples decreased significantly during storage and were significantly increased by addition of a 10% CaCl_2_ solution. The pH levels of freshly collected WB samples were 6.0–6.5, and similar pH was observed in stored WB samples. Addition of the ACD-A solution (~10%) did not significantly decrease the pH of the stored WB samples. For reference, pH of ACD-A solution was 4.5–5.0.

**Fig. 2:**
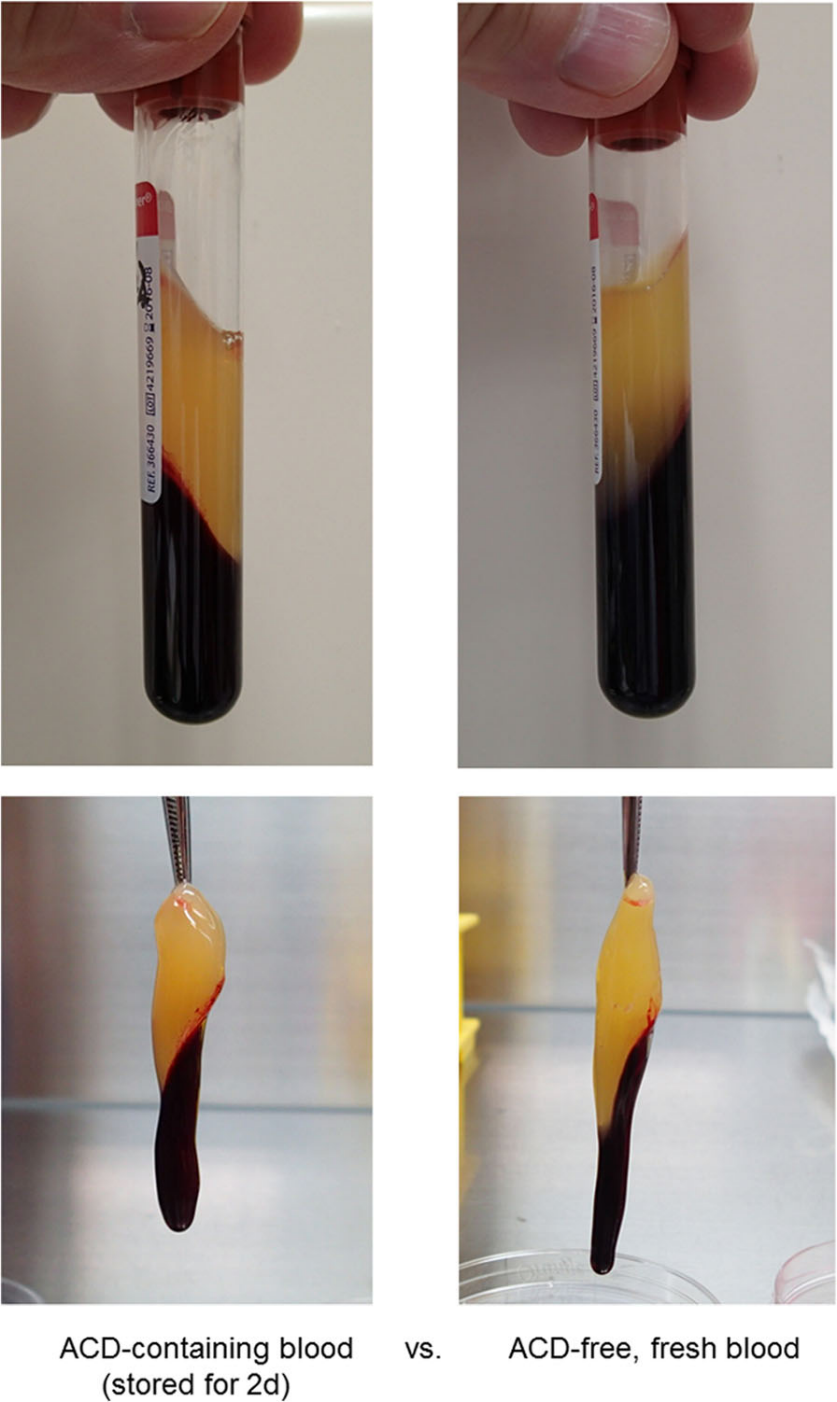
Appearance of PRF clots prepared from WB samples stored for 2 days. These observations are representative of WB samples obtained from four donors

**Fig. 3:**
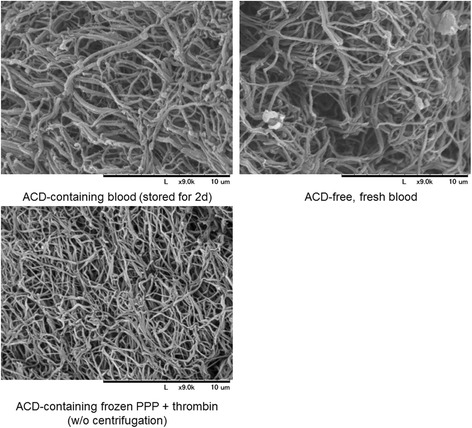
SEM examination of fibrin fibers formed in self-clotted PRF and thrombin-stimulated PPP clots. PRF was prepared from fresh and 2-day-old WB samples. Similar observations were obtained from WB samples collected from three other donors. *Scale bars* = 10 μm

The appearance of PRF clots prepared from freshly collected WB samples and WB samples stored for 2 days are shown in Fig. 2. There were no visual differences between these two PRF preparations. Microstructure of fibrin clots formed from fresh and 2-day-old WB samples is shown in Fig. 3. As for thickness and cross-links of fibrin fibers, no substantial differences were observed. For reference, fibrin clots that were prepared from PPP and bovine thrombin were composed of apparently thinner fibrin fibers as compared with PRF clots from either fresh or stored WB samples.

**Fig. 4:**
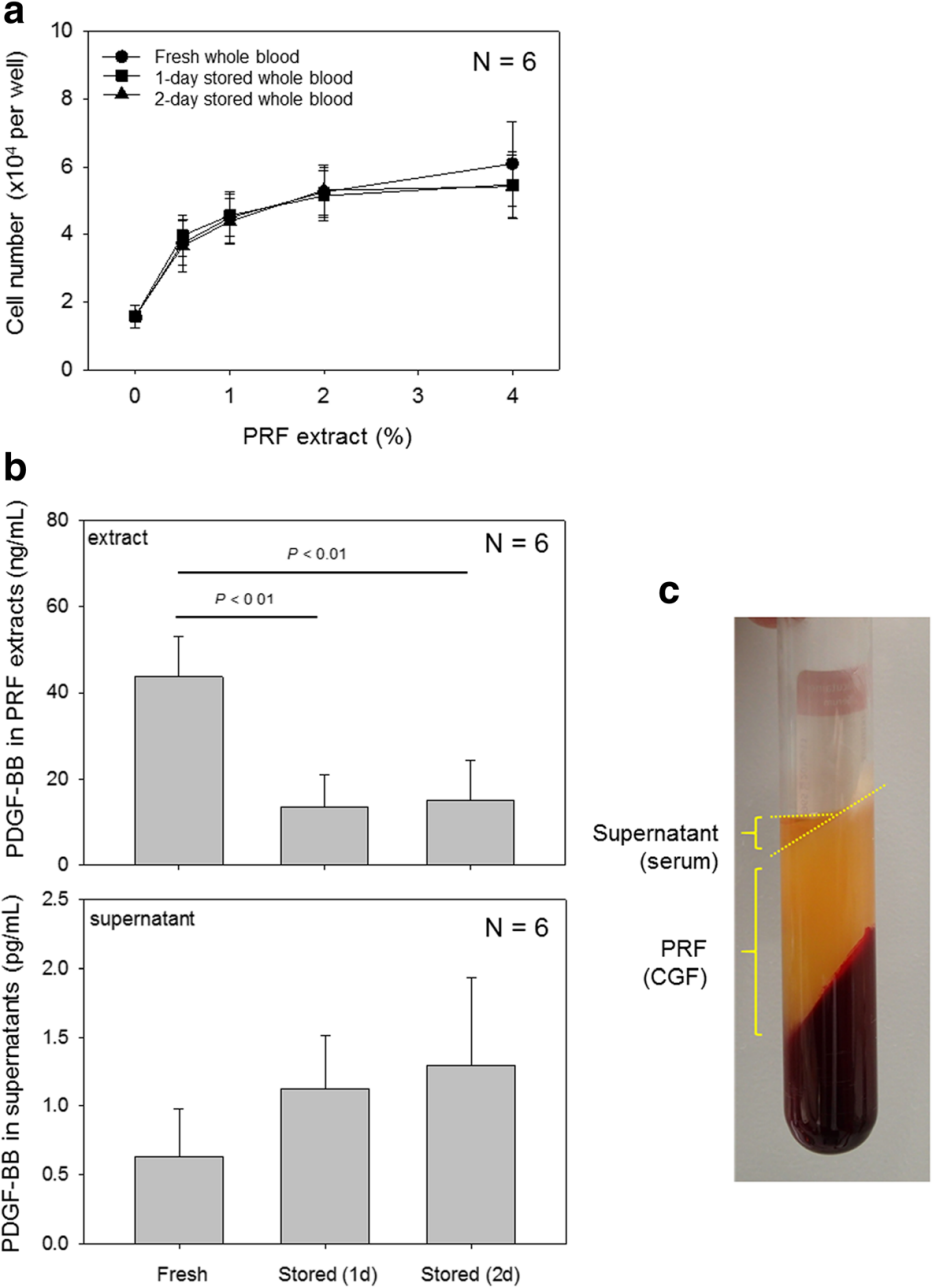
Bioactivities and PDGF-BB concentrations in PRF extracts and the supernatant serum fraction. **a** PRF extracts were added to periosteal cell cultures and incubated for 3 days to evaluate their effects on cell proliferation. No significant differences were observed among three groups. **b** PRF extracts were subjected to measurement of PDGF-BB levels using an ELISA kit. No significant differences were observed in the supernatant among three groups. *N* = 6. **c** Representative localization of supernatant serum fraction of PRF preparation just after centrifugation

The biological activity was tested on human periosteal cells. The effects of PRF extracts on the cell proliferation are shown in Fig. 4a. PRF extracts (0–4%) prepared from fresh, 1-day-old, and 2-day-old WB samples exerted similar stimulatory effects on the proliferation of periosteal cells. PDGF-BB concentrations in PRF extracts prepared from fresh and stored WB samples are shown in Fig. 4b. PRF extracts and the supernatant serum fraction (see Fig. 4c) were subjected to measurement of PDGF-BB levels. The concentration of this representative growth factor of platelet concentrates [4] was significantly reduced in PRF extracts by storage. In contrast, PDGF-BB levels noticeably (but not significantly) increased in the supernatants.

## Discussion

Platelet preservation is restricted to 3 and 5 days in Japan and worldwide, respectively. This limit is based on the fact that platelets are sensitive to changes in temperature and pH: when samples are stored at 2 to 6 °C, platelets become unsuitable for production of platelet concentrates [3]. Preservation of platelet concentrates results in a drop of pH below 6.0 depending on the platelet count [15], and pH below 6.2 correlates with decreased in vivo efficacy of platelets [16]. Furthermore, it was recently demonstrated that growth factors in PRP degrade in the course of storage at 22 °C [17].

On the other hand, in general, WB can be stored in the presence of ACD or citrate phosphate and dextrose (CPD) at room temperature for a relatively long period (3 weeks or longer) before it is processed into blood components [1]. The WB storage has also been supported by recent developments in oxygen-permeable plastic bags. Nevertheless, out of concern about bacterial contamination, the maximal storage period is restricted to 8 h in some countries [3]. To minimize and prevent bacterial proliferation, it is recommended to maintain white blood cells in WB samples during the initial 16 to 20 h of storage to digest bacteria during storage [18, 19].

Here, it is worth discussing which functional states of platelets are expected to be maintained during storage for subsequent preparation of platelet concentrates (to be used for regenerative therapy). There is no doubt that the functional states observed in freshly isolated platelets are the best for preparation of platelet concentrates and for their best clinical performance. Nevertheless, given that platelet concentrates are expected to provide significant amounts of growth factors and fibrin(ogen) at implantation sites, stored platelets are not necessarily expected to function as fully as fresh ones (e.g., in terms of aggregation). Rather, stored platelets are expected not to lose growth factors during the storage period, while coagulation factors, especially those involved in the endogenous coagulation cascade, should maintain their activities to convert and polymerize fibrinogen to form well cross-linked fibrin fibers.

Considering the current status of clinical use of platelet concentrates in the fields of periodontology and oral surgery, in this study, we used 10-mL glass tubes that are not oxygen-permeable instead of oxygen-permeable plastic bags for storage of large volumes of WB or platelets. We advanced a working hypothesis that the storage of WB samples in glass tubes would result in a more rapid and substantial pH drop and inactivation of several enzymes involved in coagulation. This study revealed that addition of an optimal amount of a CaCl_2_ solution successfully restored the coagulation ability of the anticoagulant-supplemented WB samples. The fibrin fibers prepared from the stored WB samples were almost identical to those of fresh WB samples. PDGF-BB concentrations were significantly lower in PRF extracts prepared from stored WB samples than in those of fresh WB samples. This effect can be explained by a growth factor release from platelets after stimulation by calcium ions or maybe (less likely) by degradation of PDGF-BB. Nonetheless, the bioactivities did not significantly worsen during the short storage.

In general, autologous platelet concentrates are prepared and immediately used for regenerative therapy in dental clinics at present. Our method should expand the clinical applicability of platelet concentrates, especially PRF preparations, and make the treatment schedule more flexible.

## Conclusions

The self-clotted types of platelet concentrates (PRF) can be prepared from ACD-containing stored WB by addition of CaCl_2_ without a significant reduction in their bioactivity and without other specific reagents or devices. This approach should contribute to dissemination of PRF therapy.

## Abbreviations

ACD: Acid citrate dextrose solution
CGF: Concentrated growth factors
CPD: Citrate phosphate and dextrose
EDTA: Ethylenediaminetetraacetic acid
PDGF: Platelet-derived growth factor
PPP: Platelet-poor plasma
PRF: Platelet-rich fibrin
PRP: Platelet-rich plasma
SEM: Scanning electron microscope
WB: Whole blood
